# Were there evolutionary advantages to premenstrual syndrome?

**DOI:** 10.1111/eva.12190

**Published:** 2014-08-11

**Authors:** Michael R Gillings

**Affiliations:** Department of Biological Sciences, Macquarie UniversitySydney, NSW, Australia

**Keywords:** behavioural genomics, evolutionary medicine, evolutionary psychology, human population, neurotransmitter receptors, sex hormones, women's health

## Abstract

Premenstrual syndrome (PMS) affects up to 80% of women, often leading to significant personal, social and economic costs. When apparently maladaptive states are widespread, they sometimes confer a hidden advantage, or did so in our evolutionary past. We suggest that PMS had a selective advantage because it increased the chance that infertile pair bonds would dissolve, thus improving the reproductive outcomes of women in such partnerships. We confirm predictions arising from the hypothesis: PMS has high heritability; gene variants associated with PMS can be identified; animosity exhibited during PMS is preferentially directed at current partners; and behaviours exhibited during PMS may increase the chance of finding a new partner. Under this view, the prevalence of PMS might result from genes and behaviours that are adaptive in some societies, but are potentially less appropriate in modern cultures. Understanding this evolutionary mismatch might help depathologize PMS, and suggests solutions, including the choice to use cycle-stopping contraception.

## Introduction

During the last seven to ten days of the menstrual cycle, women may suffer from a variety of unpleasant symptoms. This cluster of physical, emotional and behavioural effects was first called ‘premenstrual tension’ (Frank 1931), and later modified to ‘premenstrual syndrome’ (PMS) to better reflect the diverse symptoms and variation in severity between individuals (Greene and Dalton 1953). Because of the variability in presentation of symptoms and the reliance on self-reporting, there are often problems with the methodologies used to investigate PMS (Parlee 1973; Brown et al. 2011; Matsumoto et al. 2013).

The majority of women exhibit some symptoms during the late luteal phase of the menstrual cycle (Biggs and Demuth 2011). Some 5 to 8% of women have symptoms severe enough to be distressing and debilitating (Yonkers et al. 2008), and experience disruptions to relationships, work and social activities. The levels of disruption in severe cases are similar to those of patients with major depression (Freeman 2003). Milder symptoms affect most women, an observation dating back to the time of Hippocrates. The syndrome affects women in all countries where PMS been investigated (Ericksen 1987; Reiber 2008; Epperson et al. 2012).

Recently, there has been a concerted effort to reach a consensus on diagnostic criteria for premenstrual disorders and to identify core symptoms of the syndrome (O'Brien et al. 2011). These symptoms include anxiety/tension, mood swings, aches and cramps, cravings and disinterest in usual activities (Freeman et al. 2011). Review of the literature on PMS, and in particular the more severe form, premenstrual dysphoric disorder (PMDD), has resulted in the inclusion of PMDD as a new category for the Diagnostic and Statistical Manual of Mental Disorders fifth edition (DSM-V) (Epperson et al. 2012; Epperson 2013). However, given the significant variation in both severity and diversity of symptoms between women, it seems difficult to draw a clear boundary between PMS and PMDD. Further, inclusion in the DSM pathologizes a series of symptoms exhibited in some degree by many women, and which, by definition, should be considered normal.

When apparently maladaptive states are universal, and at high frequency in populations, it raises the question as to whether there is an evolutionary basis for their widespread nature (Kinney and Tanaka 2009). In some cases, apparently maladaptive states may be the result of our evolutionary history and the recent emergence of humans from hunter-gathering or traditional agricultural lifestyles (Baptista et al. 2008). While it is not possible to know what conditions prevailed across 200 000 years of modern human evolution, nor to definitively reconstruct the various selection pressures across that time period (Foley 1995), it is still productive to think about the modern human condition as having recently emerged from our evolutionary past (Nesse et al. 2010). Here, we examine some evolutionary explanations for the existence of PMS, and the implications that these might have for women in modern societies.

## Critiques on evolutionary perspectives of PMS

Considering the universality of PMS, there have been surprisingly few explanations for its frequency and persistence in human populations. Many studies have investigated the proximate causes of PMS, but few researchers have asked questions about the ultimate cause of the symptoms exhibited during the late luteal phase. Over the last fifty years, the following hypotheses have been put forward as evolutionary explanations for PMS:

### Increased ardour

This hypothesis suggested that PMS arose as a consequence of failure to conceive during the current cycle (Rosseinsky and Hall 1974). Female hostility towards partner males during the late luteal phase would interrupt mating opportunities, and as a result, would intensify male ardour, and lead to improved chances of fertilization at the next ovulation. Consequently, PMS would then be evolutionarily advantageous. The objection that males need merely seek out females not exhibiting PMS was originally countered by supposing that all females in a cohabiting population tended towards synchronous cycles (Stern and McClintock 1998). However, reports of this phenomenon have been criticized for their methodological and statistical flaws, and have failed to be consistently replicated (Wilson 1992; Strassmann 1997, 1999a,b,c).

### Cyclic changes in immunosuppression

The luteal phase of the menstrual cycle is characterized by elevated progesterone and oestrogen levels. This, in turn, supresses cell-mediated immunity and relaxes surveillance of infectious agents normally controlled by this arm of the immune system. Consequently, the diverse symptoms and severities of PMS have been suggested to arise from a similarly diverse suite of pathogens whose effects are exacerbated by relative immunosuppression during the luteal phase (Doyle et al. 2007). Antibiotic therapy can ameliorate PMS symptoms (Toth et al. 1988), but if infectious agents were the root cause, it is surprising that this therapy has not been widely adopted. One might also expect that the improved general health of modern populations with respect to otherwise cryptic pathogens and parasites would itself have lowered the incidence of PMS.

### Positive states during ovulation

During the fertile phase of the menstrual cycle, women experience positive physical and behavioural states that improve their chances of reproduction (Gangestad et al. 2002; Roberts et al. 2004; Haselton et al. 2007; Gangestad et al. 2010; Grammer et al. 2004). Some authors have suggested that when these positive states cease during the premenstrual phase, it leads to states of relatively lower physical and mental happiness that are ‘subjectively experienced as symptoms’ characteristic of PMS (Reiber 2008). This hypothesis does not explain why symptoms of PMS abate at menstruation, or why women do not exhibit PMS symptoms at all times other than when ovulating.

### Diversion of metabolites

This hypothesis suggests that the metabolic costs of menstruation divert glucose from the brain to reproductive functions (Gailliot et al. 2010). The diversion then leads to a changes to processes in the brain that regulate self-control, leading to the ‘symptoms of impulsivity’ that characterize PMS (Bröder and Hohmann 2003; Pearson and Schipper 2009). However, this hypothesis is difficult to reconcile with observations that adiposity increases the risk of PMS (Bertone-Johnson et al. 2010), as increased fat reserves should buffer metabolic deficiencies, and such deficiencies could also simply be addressed by increased calorific intake. Given that the premenstrual phase is characterized by decreases in circulating hormones, and that the next cycle of ovarian and uterine development has not been initiated, there does not seem to be any clear reason why metabolic costs should increase differentially during the premenstrual phase.

### Sexual and relationship rejection

Ongoing bonding between humans is complex, depending on sexual and nonsexual behaviours, and on previous experience in the relationship. Where such relationships do not result in pregnancy, premenstrual hostility may cause varying degrees of rejection, both sexually and of the relationship in more general terms. It might then be conjectured that infertile pair bonds are more likely to break down, freeing both partners to pursue fertile mates (Morriss and Keverne 1974).

Each of these hypotheses provides a potential explanation for the potentially maladaptive spectrum of symptoms that comprise PMS. However, only the first and last explanations are evolutionary, in the sense that they encompass scenarios that have the potential to alter reproductive outcomes and thus be subject to evolution by natural selection. Of course, it is still possible that PMS may be the result of a trade-off, or pleiotropy, and is associated with some other, as yet unidentified fitness benefit.

Nevertheless, for the purposes of this paper, we would argue that the frequency of PMS in human populations suggests it may have had selective advantages that maintained the phenomenon at high frequencies. Explanations that invoke sexual or relationship breakdown may explain such advantages. Further, seeking to understand the ultimate cause of PMS allows us to view it from a different, and perhaps more constructive perspective, and might suggest means for modifying unwanted symptoms.

If relationship breakdown was the ultimate evolutionary advantage conferred by PMS, a number of predictions might be made. Firstly, PMS should be heritable, and at least some loci should exhibit genetic variants that are associated with the syndrome. Secondly, hostile behaviours associated with PMS should be preferentially directed at current partners. Thirdly, behaviours and physiology associated with PMS should increase the possibility of forming new pair bonds. Examination of these proximate effects does indeed confirm the predictions.

## Genetic variation and PMS

The severity of PMS and PMDD is linked with a sensitivity to cycling concentrations of oestrogen and progesterone (Schmidt et al. 1998), and this in turn appears to have a genetic basis. Genetic influences on premenstrual symptoms have been investigated using twin studies in Australia (Condon 1993; Treloar et al. 2002), the USA (Kendler et al. 1998) and the UK (Van Den Akker et al. 1995), yielding estimates of heritability ranging between 30 and 80%. Recent quantitative genetic modelling using Malaysian and Iranian twins suggested that additive genetic variation accounted for 95% of the observed variability in PMS symptoms, while unique environmental factors accounted for the remaining 5% (Jahanfar et al. 2011). Together, these studies demonstrate genetic control over PMS. The general consensus is that the differences in severity and range of PMS symptoms are due to variation at a number of gene loci (Condon 1993; Kendler et al. 1998; Treloar et al. 2002).

Hypotheses about the variable phenotypic expression of PMS and PMDD usually invoke a particular sensitivity to the variation in the cycling concentrations of hormones (Mortola 1996; Cunningham et al. 2009; Biggs and Demuth 2011), because the absolute levels of progesterone, oestrogen and testosterone do not differ between individuals at high or low risk of PMS (Bäckström et al. 1983). Consequently, it has been suggested that PMS arises through an interaction between hormones and receptor or neurotransmitter variants (Mortola 1996; Dickerson et al. 2003). Likely candidate loci include those with roles in reception of oestrogen and progesterone (Cunningham et al. 2009; Biggs and Demuth 2011), or genes for neurotransmitter receptors, particularly serotonin, as intermittent use of selective serotonin reuptake inhibitors can ameliorate the symptoms of PMS (Dimmock et al. 2000; Brown et al. 2009; Pearlstein 2012).

Advances in human behavioural genomics have begun to examine the role of genetic polymorphisms in the aetiology of PMS. Variants in the oestrogen receptor alpha gene, more particularly in intron 4, are associated with risk for PMDD, supporting the notion that genes dealing with reproductive steroids may be involved, at least in the Caucasian population investigated during this study (Huo et al. 2007). Further study of particular single nucleotide polymorphisms may allow more reliable associations with the individual traits of PMDD (Miller et al. 2010), but larger cohorts and examination of more loci will be needed.

Evidence suggests that falling levels of ovarian hormones in the luteal phase, particularly of oestrogen, may affect the activity of central serotonin in susceptible individuals. Consequently, polymorphisms in genes regulating serotonergic activity have been a popular target in studies to elucidate a genetic basis for PMS (Dhingra et al. 2007). Analyses of polymorphism in the serotonin 1A receptor gene (HTR1A) show that PMDD is associated with the G/G HTR1A (rs6295) polymorphism. This has led some authors to suggest that consequent declines in serotonergic neurotransmission might be responsible for premenstrual changes to working memory and cognitive control in women with PMDD (Yen et al. 2013).

Variants in the promoter for the serotonin transporter gene have effects on expression of the serotonin 5-HT transporter molecule. Promoter variants are associated with neuroticism, depression, seasonal affective disorder and perhaps with PMDD (Praschak-Rieder et al. 2002), although this conclusion must be regarded as preliminary, as results have not been replicated (Dorado et al. 2007). In general, the results of association studies must be treated with some caution, as they cannot prove causal relationships, and because of practical restrictions on sample size, they often have poor statistical power (Yen et al. 2013).

Other neurotransmitter pathways have also been investigated. Polymorphisms in the steroid-5-alpha-reductase, alpha polypeptide 1 gene may protect women against severe PMS (Adams and McCrone 2012). Reduced bioavailability of brain-derived neurotrophic factor (BDNF) in humans can be caused by a known polymorphism in the BDNF gene in humans. When the human mutation was inserted into the corresponding mouse gene, female mice demonstrated increased anxiety-like behaviours specifically during the low oestrogen phases of the menstrual cycle (Bath et al. 2012). The frequency of human homozygotes for the Valine66Methionine polymorphism implicated in this study is 4%, similar to the frequency of PMDD in human populations.

It is likely that the genetic determinants of PMS and PMDD involve multiple loci that affect the production, transport and reception of sex hormones and neurotransmitters. Identifying genetic polymorphisms with major roles in the expression of these syndromes will require larger cohorts of subjects and more intensive DNA sequencing efforts. Animal models are unlikely to be appropriate for PMS studies, although use of such models may be one method to establish causal relationships between genetic polymorphisms and PMS.

## Is PMS directed at current partners?

If PMS has been selected on the basis that it increases the probability that women will dissolve infertile partnerships, then it follows that the animosity exhibited during PMS should be preferentially directed towards current partners. There is some evidence that this is the case. PMS sufferers score more highly on measures of family conflict (Kuczmierczyk et al. 1992), and the heightened sensitivities caused by PMS were greatest at home in a study of women in the USA, UK and France (Hylan et al. 1999). There is a significant relationship between menstrual distress and marital dissatisfaction (Coughlin 1990). Marital relationships of PMS sufferers deteriorate during the luteal phase, whereas relationship satisfaction is similar between PMS and nonPMS sufferers during the follicular phase (Ryser and Feinauer 1992). Of course, male behaviours are also part of this equation, and it is known that male partners often respond to premenstrual symptoms by avoidance and withdrawal (Cortese and Brown 1989), increasing the likelihood of estrangement. Comparisons between the quality of life in the follicular versus luteal phases of PMS sufferers show the greatest relative decline in attitudes towards immediate family and marital status (Halbreich et al. 2003). The monthly conflict associated with PMS has been linked to deterioration of relationships and to divorce (Graze et al. 1990), although the wide variety of outside factors to be considered make demonstration of causality difficult. The preferential direction of PMS towards partners is suggested by the fact that conjoint monitoring of PMS symptoms within a relationship improves marital satisfaction (Frank et al. 1993). Consequently, it does appear that PMS symptoms are more extreme in the home, that animosity is directed at partners, and that marital dissatisfaction peaks during the luteal phase for PMS sufferers. Together, these phenomena might increase the likelihood of partnerships dissolving.

There are significant costs to dissolving pair bonds, in terms of the potential failure to find a new mate, and the loss of paternal investment in existing offspring, such that on balance, dissolution may only be beneficial in pair bonds with no offspring. Under such circumstances, it would also be possible for females to pursue extra-pair copulation to overcome the infertility of their partner, while remaining in their current pair bond (see below). However, even if small proportions of women in infertile relationships took up new, fertile partners as a result of PMS, this would be sufficient to select and maintain PMS in populations (Frankham et al. 2010).

## Does PMS increase the likelihood of changing partners?

If PMS evolved because it improved the chances of women dissolving infertile partnerships, then behaviours exhibited during PMS should enhance the likelihood of forming a new pair bond. Most women feel more sexual at particular phases of the menstrual cycle, and the premenstrual phase is nominated by more women than any other phase (Harvey 1987), despite the physical inconvenience associated with premenstrual symptoms. The literature does report some contrary findings, but a review of ten studies shows an increase, or at least no decline in sexual activity during the luteal phase (Hill 1988).

Finding new partners involves a degree of risk and intrasexual competition. Risk taking varies across the menstrual cycle, with women being most risk-averse during ovulation, and exhibiting more risky behaviours during nonfertile phases (Chavanne and Gallup Jr 1998; Bröder and Hohmann 2003). Women also display more competitive behaviours in the premenstrual phase in a number of experimental scenarios (Pearson and Schipper 2009; Buser 2012). PMDD is associated with increased activity in the amygdala during the late luteal phase. Some researchers suggest that changes in limbic activity lead to a consequent increase in impulsivity (Protopopescu et al. 2008).

The premenstrual phase ends with the onset of menstrual bleeding. In many animals, the uterine lining is resorbed, thus conserving the proteins and iron assigned to the endometrium in preparation for implantation. Amongst the primates, humans and chimpanzees are unusual, in that they exhibit copious external bleeding. This is hard to understand, given the potential nutritional costs involved, and several hypotheses have been put forward to account for the phenomenon. Suggestions that menstruation protects against sperm-borne pathogens (Profet 1993; Howes 2010) have been criticized (Strassmann 1996; Finn 1998), and these latter authors, respectively, suggest energy conservation and pleiotropy as alternative explanations.

It is clear however, that the external evidence of menstruation is a signal of impending fertility, and thus may advertise this condition to other males. If this were the case, one might expect males to cloister their partners in a form of mate guarding that conceals the female's reproductive status (Strassmann 1992). Indeed, it has recently been suggested that the taboos surrounding menstruation that are promulgated by the major religions of the world are in place as a protection against cuckoldry (Strassmann et al. 2012).

## Consequences of the hypothesis

Under this hypothesis, the frequency of PMS and PMDD in modern populations arises because of a mismatch between our evolutionary history and current cultural conditions (Baptista et al. 2008). While PMS could still function to dissolve infertile partnerships in modern societies, most PMS symptoms in developed nations arise as a consequence of our control over reproduction, not as a consequence of infertility. Women in hunter-gatherer societies were likely to be pregnant, or if caring for an infant, in a state of lactational amenorrhoea for most of their reproductive lives, and would consequently experience fewer menstrual cycles. Studies of modern tribespeople with hunter-gatherer lifestyles show that fertile women have a median of two menstrual cycles per year and just over 100 per lifetime. This compares with women in developed countries who are likely to have over 400 cycles (Short 1976, 1994; Strassmann 1997, 1999a,b,c).

For individual women, constant cycling involves the loss of proteins, sugars and iron on a regular basis, and causes a suite of unpleasant physical symptoms and possible psychological distress. A woman with PMDD is likely to have almost 3000 days with symptoms during her reproductive life (Rapkin and Winer 2009). Regular cycling is also associated with increased risk of uterine, breast and endometrial cancers (Strassmann 1999a,b,c; Gladwell 2000). For couples, PMS places stress on relationships, and for society it has economic costs (Dean and Borenstein 2004).

The frequency of menstruation in modern humans may be maladaptive. This situation most probably arises because patterns of reproductive cycling in modern cultures are evolutionarily recent, but we still carry the genetic toolbox of our hunter-gatherer and agricultural ancestors. There is a solution to this dilemma, suggested many times in the literature. The adoption of cycle-stopping contraception would mimic our ancestral state (Strassmann 1999a,b,c; Thomas and Ellertson 2000). It would have the additional benefits of ameliorating the symptoms of PMDD (Halbreich et al. 2003) and lowering the incidence of some reproductive cancers (Schindler 2013). However, it should be noted that the use of hormonal contraception can alter mate choice and thus may also affect reproductive outcomes (Alvergne and Lummaa 2010).

## Conclusion

Inclusion of severe premenstrual symptoms as a full category in the *Diagnostic and Statistical Manual of Mental Disorders* (DSM-V) may encourage more research on PMDD and PMS (Epperson et al. 2012). However, it does continue to stigmatize PMS and PMDD as being disease states (Ussher and Perz 2013). Under the hypothesis we have proposed, PMS and PMDD are not diseases or ‘syndromes’, but arise as a normal consequence of adaptive strategies developed during our evolutionary history, similar to morning sickness (Flaxman and Sherman 2000) and other apparently maladaptive states (Baptista et al. 2008; Kinney and Tanaka 2009).

There are some clear predictions arising from this hypothesis. The probability of relationship dissolution should vary with menstrual phase. One might also expect that early reproduction would ameliorate the intensity of PMS, and conversely, PMS symptoms might intensify over time in pair bonds with no offspring. There should be an increased frequency of dissolution of infertile pair bonds amongst human societies whose reproductive cycles are similar to the proposed ancestral condition. It will be difficult to disentangle all the potential drivers of these effects, especially given the diverse influences of environment and culture, and carefully designed twin studies might be needed. The diversity and varying severity of PMS symptoms are probably explained by the contributions of many genes, each with multiple alleles. It is important that the search for such proximate causes of PMS symptoms does not overshadow the ultimate cause, which may be to maximise reproductive fitness.
